# Rabbit-Derived Antithymocyte Globulin-Associated Perioperative Anaphylaxis in Renal Transplantation: A Multidisciplinary Perspective on Pathophysiology, Clinical Presentation, and Management

**DOI:** 10.3390/antib14040092

**Published:** 2025-10-28

**Authors:** Imran Gani, Usman Baig, Ahmad Mirza, Shais Jallu, Abrar Ahad Chawdhary

**Affiliations:** 1 Department of Nephrology, Hypertension and Transplant Medicine, Wellstar MCG Health, Augusta University, Augusta, GA 30912, USA; 2 Independent Researcher, Atlanta, GA 30318, USA; 3 Transplant Surgery Division, Wellstar MCG Health, Augusta University, Augusta, GA 30912, USA; 4 Department of Pulmonary and Critical Care Medicine, Saint Luke’s Hospital of Kansas City, Kansas City, MO 64111, USA; 5 Department of Anaesthesia and Operations, College of Applied Medical Sciences—Khamis Mushait, King Khalid University, Abha 61421, Saudi Arabia

**Keywords:** thymoglobulin, anaphylaxis, transplantation, allergy, shock, rabbit, rATG

## Abstract

Rabbit antithymocyte globulin is one of the most commonly used agents for induction immunosuppression in renal transplantation. It has contributed significantly to improved allograft survival and has a favorable safety profile. Despite its advantages, rabbit antithymocyte globulin carries a rare but potentially life-threatening risk of anaphylaxis, which can lead to severe morbidity and mortality. Anaphylaxis is an acute and dramatic complication that requires prompt recognition and immediate management. In this review, we discuss the pathophysiology, clinical features, and management of rabbit antithymocyte globulin-associated anaphylaxis. We have also included practical insights from our clinical experience to guide early recognition and management, aiming to help clinicians safely manage this critical adverse event.

## 1. Introduction

Induction immunosuppression is one of the cornerstones in renal transplantation, which is crucial for preventing early transplant rejection. It not only improves outcomes in the early period, but has also been associated with improved graft survival rates in the long term, and facilitates transitioning to maintenance immunosuppression [1,2]. Early protocols included the use of corticosteroids and Muromonab-CD3 which were associated with broad immunosuppression and significant side effects [3]. Newer agents, including basiliximab, alemtuzumab, and rabbit antithymocyte globulin (rATG), have a better safety profile and selection can be tailored according to the risk status of the patient and center preference [1,3,4].

The rATG consists of polyclonal antibodies against T lymphocytes, and was initially approved only for the use of acute cellular rejection [3]. It is currently one of the most commonly used induction immunosuppressive agents in the U.S., and its use is estimated in more than 50% of the cases [5,6,7]. It was approved by the U.S. Food and Drug Administration (FDA) in 2017 for induction immunosuppression; however, it has already been widely used in clinical practice for over four decades [4,7]. In comparison with monoclonal antibodies, it has lower rates of acute rejection, graft loss, and death at five years post-transplant [7]. In addition, it can also be used in high-risk patients, such as those with high panel reactive antibodies, HLA mismatches, or those with a prior history of transplantation [8].

The rATG is generally well tolerated, with common side effects including cytopenias and infusion site reactions [9]. However, anaphylaxis is a rare but potential complication following rATG infusion. Only a few cases of anaphylactic reactions have been reported in the literature, most of which led to the postponement of transplant surgery [10]. Further, anaphylaxis may also occur during the use of rATG for acute renal allograft rejection. Such reactions are not limited to renal transplantation but have also been observed with its usage in other solid organ transplantation [11].

Although anaphylaxis is rare, early recognition is essential, as it can be mistaken for conditions like cytokine release syndrome and anaphylactoid reaction [12,13]. Given its life-threatening nature and the risk of progressing to refractory anaphylaxis, immediate and appropriate treatment is critical. In transplant surgery, timely intervention becomes even more crucial, as clinical deterioration and delayed recovery could affect the outcomes of transplantation. Moreover, anaphylactic shock can lead to extended hospital stays, increased adverse outcomes, and higher healthcare costs [14].

The purpose of this literature review is to review the pathogenesis, clinical presentation, management, and diagnostic evaluation of thymoglobulin-associated anaphylaxis in renal transplant recipients. It will also include recommendations based on our clinical experience, which will offer insight into multidisciplinary care.

## 2. Pharmacology

It is important to have a clear understanding of the pharmacology of antithymocyte globulins (ATG). This can help explain the mechanisms behind the side effects and help with devising a management strategy.

### 2.1. Manufacturing

**Figure 1:**
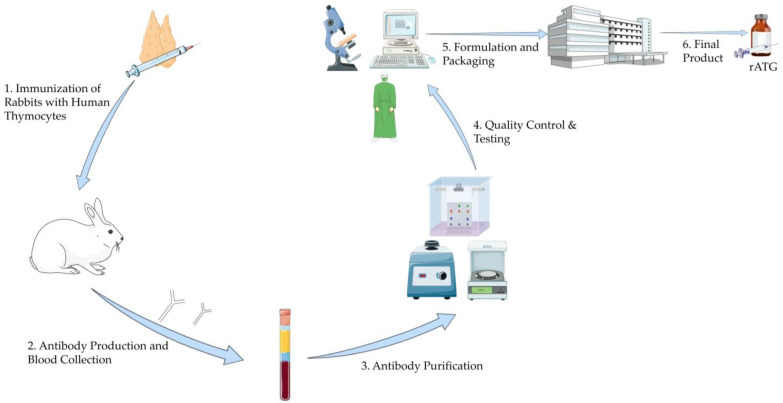
Manufacturing Process of Rabbit Antithymocyte Globulin. This illustration was created using resources from Servier Medical Art (licensed under CC BY 4.0). Available at: https://smart.servier.com (accessed on 5 June 2025). Abbreviation: rATG: Rabbit Antithymocyte Globulin.

Commercially, there are three formulations of antithymocyte globulins: rATG, horse-derived ATG (Atgam), and porcine-derived ATG. The first two are available in the U.S., while the porcine-derived ATG is only available in China [15,16]. ATGs are produced by immunizing the aforementioned animals with human thymocytes, which stimulates the production of antithymocyte antibodies. After immunization, plasma is collected, and the IgG fraction is isolated and purified to remove contaminants. The final product undergoes further evaluation to ensure its safety and immunosuppressive properties (Figure 1) [17].

### 2.2. Clinical Profile Based on the Type of Antithymocyte Globulins

Among rabbit-derived and horse-derived ATG, rATG is clinically superior. In a randomized controlled trial conducted by Brennan et al. to assess the efficacy and safety of rATG compared to Atgam for induction therapy in adult renal transplant recipients, rATG demonstrated superior outcomes [18]. It was associated with a significantly lower incidence of acute rejection at one year. The severity of rejection (as per the Banff Criteria) was also lower with rATG. In addition, adverse side effects were low with rATG. At the five-year follow-up of these patients, rATG was associated with higher event-free survival, improved graft survival, and greater freedom from rejection [19]. Alongside these improved outcomes with rATG, it is also associated with a decrease in healthcare costs compared to Atgam [20].

It is essential for a clinician to know the source of ATG derivation, as a pre-existing allergy to these animals could trigger an anaphylactic reaction. For the purpose of this review, we will mostly focus on rabbit-derived ATG, as it is the most frequently used form of these globulins in clinical practice.

### 2.3. Mechanism of Action of rATG

The mechanism of rATG-induced immunosuppression occurs through multiple interconnected pathways which mainly target T-cell populations, inducing broader immunomodulatory effects, and also by influencing other immune cells, as outlined below:

1. T cells: Polyclonal composition enables broad targeting of T-cell surface markers, such as CD2, CD4, CD8, and TCR. Depletion of T cell levels occurs by complement-mediated lysis, apoptosis induction, opsonization, and phagocytosis. This effect is not limited to peripheral lymphocytes but also involves secondary lymphoid tissue [21].
2. B cells: Although primarily a T-cell depleting agent, rATG also induces B-cell apoptosis by cross-linking surface receptors such as CD30, CD95, and CD80. The Fab fragment of the antibody plays a significant role in mediating this apoptotic process. Other mechanisms include caspase-dependent apoptosis, cathepsin B-mediated pathways, and lysosomal cysteine protease pathways [22].
3. Natural killer (NK) cells: The administration of rATG leads to profound suppression of NK cells and their cytotoxic activity, occurring at significantly lower doses compared to those required to affect other immune cell populations [23]. This suppression is mediated primarily through the binding of the Fc portion of rATG to the low-affinity IgG receptor CD16 (FcγRIII) on CD56^dim^ NK cells. Engagement of CD16 results in (i) down-modulation of CD16 surface expression, (ii) induction of apoptosis and necrosis at low antibody concentrations (as little as 0.1 µg/mL), and (iii) functional impairment, including reduced degranulation, interferon-γ production, and cytotoxicity against target cells. These effects are largely restricted to the CD56^dim^ NK subset, which is the principal effector population for cytotoxicity [23].
4. Plasma cells: Plasma cell levels also decrease, which could be attributed to changes in T-cell subset (depletion of T-follicular helper cells) [24].

### 2.4. Dosage and Administration

The rATG for induction immunosuppression is usually administered at the rate of 1.5 mg/kg for 3–7 days based on center preference and the patient’s risk factors for rejection [7]. It may be given either before or after the start of surgery [7]. rATG can be administered via a central line (jugular access) or through peripheral access after appropriate premedication is administered, with specific practices varying by transplant center [25]. Clear communication among all team members is important once induction has begun, particularly to look out for potential adverse effects.

### 2.5. Side-Effects of rATG

The side effects of rATG range from mild reactions such as fever, urticaria, and vomiting to severe complications like disseminated intravascular coagulation and anaphylactic shock [9,24,26,27,28]. A list of side effects is presented in Table 1.

## 3. Rabbit-Derived Antithymocyte Globulin-Associated Anaphylaxis: Pathophysiology and Clinical Presentation

Perioperative anaphylaxis occurs in approximately 1 in 10,000 to 1 in 20,000 cases, with the exact incidence in transplant surgeries unknown. However, it likely has an even lower incidence in this context [29]. Despite its low occurrence, it remains a significant concern when encountered. Perioperative anaphylaxis has been reported after the administration of neuromuscular blocking agents, latex exposure, and antibiotics such as cefazolin [13,30].

### 3.1. Diagnostic Criteria

Anaphylaxis is typically diagnosed using one of two criteria: the National Institute of Allergy and Infectious Diseases (NIAID) criteria or the World Allergy Organization (WAO) criteria. The NIAID criteria, established in 2006, require the presence of skin and/or mucosal manifestations. In contrast, the WAO criteria, established in 2020, are relatively newer and recognize that anaphylaxis can occur even in the absence of skin or mucosal symptoms [31,32]. A simplified version of both criteria is presented in Table 2. In a study conducted by Yeğit et al. in Türkiye, comparing the WAO criteria with other commonly used criteria, the WAO criteria were able to diagnose an additional 6% of patients [33].

We also recommend using the WAO criteria because it recognizes that anaphylaxis can occur without cutaneous manifestations, which has been reported in 10 to 20% of cases [32]. Also, its simplicity makes it easier to use in the operating room, where multiple diagnostic challenges may arise, making timely diagnosis difficult. These challenges will be discussed further in the next sections.

### 3.2. Mechanisms of Anaphylaxis

**Figure 2:**
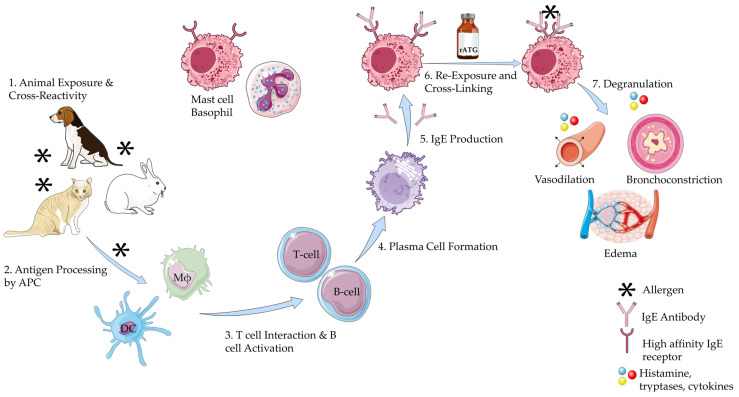
Mechanism of Anaphylaxis to Rabbit Antithymocyte Globulin. This illustration was created primarily using resources from Servier Medical Art (licensed under CC BY 4.0). Three specific components were adapted from NIAID NIH BIOART: Plasma B cell: Illustration from NIAID NIH BIOART Source (bioart.niaid.nih.gov/bioart/409), Mast Cell: Illustration from NIAID NIH BIOART Source (bioart.niaid.nih.gov/bioart/335), Receptor Protein: Illustration from NIAID NIH BIOART Source (bioart.niaid.nih.gov/bioart/438). All remaining visual components were adapted from Servier Medical Art. Available at: https://smart.servier.com (accessed on 5 June 2025). Abbreviations: APC: Antigen Presenting Cell; DC: Dendritic Cell; Mϕ: Macrophage; rATG: Rabbit Antithymocyte Globulin.

The mechanism of anaphylaxis is mediated by the degranulation of mast cells and basophils. The initial step involves sensitization of these immune cells to an allergen, such as rabbit allergen. The allergen is processed and presented by the antigen-presenting cells, which activate T helper cells. This leads to the production of IgE antibodies by B lymphocytes, which then bind to the surface of mast cells. Upon subsequent exposure to the allergen, such as rATG (which exhibits cross-reactivity with rabbit allergens), crosslinking of the IgE receptors occurs, triggering degranulation of the mast cells and basophils [34]. During degranulation, various mediators are released, including histamine, prostaglandins, cytokines, and leukotrienes. These mediators are responsible for vasodilation, bronchoconstriction, and other immediate manifestations of anaphylaxis, leading to the uniphasic anaphylaxis reaction seen in most cases (Figure 2) [35,36,37]. Clinical evidence supports this mechanisms: for example, Brabant et al. reported a young man with documented rabbit allergy who developed anaphylaxis within minutes of rATG infusion, underscoring the role of IgE-mediated sensitization [38].

In a small subset of patients with immediate anaphylaxis, symptoms initially resolve after treatment but can reoccur after a delay, which may range from 1 to 72 h [39]. This recurrence is known as biphasic anaphylaxis. The exact mechanism underlying biphasic reactions is unclear, but it is believed to involve the activation of biochemical pathways that lead to the production of platelet-activating factor and tumor necrosis factor following the initial phase [40,41]. Initially, it was thought that biphasic reactions were primarily associated with orally consumed allergens, which provide sustained immune stimulation [39]. However, similar reactions have also been observed with parenteral agents, such as iodine-based contrast media [42]. Although biphasic anaphylaxis has not been reported in the literature with the use of intravenous (IV) rATG, it remains a theoretical possibility that clinicians should be aware of.

### 3.3. Risk Factors for Anaphylaxis

The risk factors for anaphylaxis associated with rATG primarily involve prior sensitization to rabbits, polysensitization to multiple allergens, and contamination of rATG.

In most reported cases, exposure to rabbits has occurred through domesticated pets, as they are the third most common pet in the U.S. [43]. A study by Liccardi et al. investigated the frequency of rabbit allergen sensitization among patients in an outpatient setting. Out of 753 patients who tested positive on skin prick tests, 20 were positive for rabbit dander, and 18 of these (90%) had a history of rabbit contact [44]. This shows the potential impact of having rabbits as pets, which could sensitize future transplant recipients. People working in laboratories where rabbits are used may become sensitized to rabbit allergens. Sensitization to rabbit dander can also occur in people who have no direct contact with rabbits [44].

The proteins most commonly implicated in rabbit-related allergies are rabbit serum albumin (*Ory c 6*) and other *Ory c* proteins [45]. Rabbit serum albumin is found in the rabbit blood and, to a lesser extent, excreted in urine [45]. Other *Ory c* proteins are primarily present in saliva, urine, and fur. Skin prick tests used for diagnosing rabbit allergies typically assess IgE antibodies against epithelial and urinary proteins derived from rabbits; however, these tests are not specific to individual proteins [46]. Sensitization to one or more of these specific proteins may play a role in triggering anaphylaxis during rATG administration. This area highlights a notable gap in existing literature and could be of significant interest for future research, as identifying specific allergen sensitivities could help in tailoring immunosuppressive therapy.

Polysensitization may contribute to hypersensitivity reactions to rATG. Sensitization to multiple animal allergens can develop by mechanisms of cross-reactivity. Serum albumins and lipocalins can cross-react with similar proteins from other mammals, leading to unexpected allergic reactions [47,48]. This cross-reactivity may explain positive rabbit skin prick test results in individuals without known rabbit exposure. This could also explain cases of rATG-induced anaphylaxis in the absence of prior contact with rabbits.

Contamination of rATG, though very rare, can occur during the manufacturing process and may result in anaphylaxis. In such cases, all patients receiving rATG from the affected batch would be at risk for anaphylaxis. Hill et al. reported a case of contamination in a batch of vincristine, which led to anaphylaxis, even resulting in cardiac arrest in some cases. The potential contamination was detected using mass spectrometry [49].

In addition to ATG being the active component of the infusion, the formulation also contains other ingredients such as glycine, sodium chloride, and mannitol [50]. These substances, known as excipients, improve the solubility, stability, safety, and efficacy of the medication [51]. There has been a reported case of anaphylaxis attributed to the mannitol excipient during a liver transplantation procedure [11]. Therefore, these excipients could also be a potential trigger for anaphylactic reactions.

### 3.4. Clinical Manifestations

The manifestations of anaphylaxis can range from localized skin reactions to involvement of multiple organ systems. When the respiratory system is affected, it may lead to symptoms such as difficulty breathing, wheezing, and stridor. Gastrointestinal involvement can cause diarrhea and abdominal pain. Cardiovascular involvement may result in a significant drop in blood pressure, which may lead to cardiovascular collapse.

The severity of anaphylaxis can be classified using either the Mueller or Ring and Messmer classification system [52]. Severity tends to be greater in older adults, individuals with pre-existing cardiac conditions, males, those experiencing psychological stress, and patients taking beta-blockers or ACE inhibitors [53,54]. These risk factors are commonly present in patients undergoing renal transplantation, which may explain the more severe clinical presentations observed.

Intraoperative findings following the onset of anaphylaxis include hypotension, tachycardia, hypoxia, wheezing, and angioedema [55]. The monitors may reveal significant blood pressure changes, measured either invasively or non-invasively, as well as relevant electrocardiographic (EKG) abnormalities, decreased oxygen saturation, capnographic changes, and alterations in respiratory parameters. Capnographic findings may include a drop in end-tidal CO_2_; a cutoff of 25 mmHg has a sensitivity and specificity greater than 90% in patients with anaphylaxis having severe hypotension [56]. Ventilator parameters commonly show changes associated with airway obstruction, which include increased peak inspiratory and plateau pressures, and a flattened inspiratory flow curve [57,58].

Several published cases illustrate the spectrum of clinical severity associated with rATG-induced anaphylaxis. Kandil et al. reported a 39-year-old woman who developed pulseless electrical activity within a few minutes of infusion, requiring CPR and resuscitation [59]. Navas-Blanco et al. similarly described abrupt cardiovascular collapse with airway edema, necessitating aborting the procedure despite intensive resuscitation [60]. In contrast, Raval et al. documented successful stabilization and completion of transplantation after the patient developed severe bronchospasm and hypotension, with rATG cautiously reintroduced in diluted form [61]. Fatal outcomes have also been reported, including the case by Rafat et al., who experienced refractory hypotension with patient death postoperatively, and Pyar et al., who suffered cerebrovascular complications and multi-organ dysfunction leading to death [62,63]. The clinical summary of reported cases of anaphylaxis associated with rATG administration is presented in Table 3 [10,38,59,60,61,62,63,64].

Outcomes extracted from the individual case reports (Table 3) are summarized in a structured format in Table 4.

Diagnosing anaphylaxis after the induction of anesthesia is challenging due to the masking of signs and symptoms by anesthesia, the surgical stress response, and physical barriers. Also, confusion with surgical complications can further obscure the diagnosis. Physical barriers such as surgical drapes and towels can conceal key physical findings like hives and angioedema. The use of antihistamines and corticosteroids prior to administration of the immunosuppression induction agent may also blunt the cutaneous manifestations of anaphylaxis [65]. Common surgical complications like hemorrhage can cause vital sign changes, such as hypotension and tachycardia, that can resemble those seen in anaphylaxis. Similarly, aspiration can produce respiratory symptoms and capnographic findings that may resemble those seen in bronchospasm secondary to an anaphylactic reaction.

We recommend that the surgical and anesthesia teams maintain open communication and clearly communicate when rATG has been administered. A temporal association between changes in vital signs and rATG administration is a strong indicator of anaphylaxis, requiring immediate management.

## 4. Management of rATG-Associated Anaphylaxis

The management of rATG-associated anaphylaxis involves prompt identification and differentiation from other intraoperative emergencies, followed by immediate treatment of an anaphylactic episode.

### 4.1. Differentiating Anaphylaxis from Other Intraoperative Emergencies

The management of rATG-associated intraoperative anaphylaxis involves identification and ruling out other conditions that may present with similar clinical manifestations. These conditions include pneumothorax, hemorrhage, pulmonary embolism, hemolytic transfusion reaction, and high spinal block.

Jugular venous access is commonly performed as part of standard surgical practice at many institutions and may also be used for the administration of rATG during surgery. The incidence of pneumothorax associated with this procedure ranges from 1% to 6.6% [66]. The pleura may be inadvertently punctured, which can present with hypotension, decrease in oxygen saturation, and elevated airway pressures, thus mimicking anaphylaxis. Differentiation can be made by assessing for absent or minimal breath sounds on the side of the puncture, abnormal chest movement, hyperresonance on percussion, and tracheal deviation [67]. A portable chest X-ray or point-of-care ultrasound (POCUS) should be obtained to rule out iatrogenic pneumothorax. A POCUS is a valuable and reliable tool for quick identification of intraoperative pneumothorax, offering advantages over traditional methods like chest x-ray, as it allows for real-time bedside assessment, thus facilitating a prompt diagnosis and treatment. Chest x-ray still remains an alternative in case the ultrasound is not available. If diagnosed, needle decompression or placement of a chest tube should be performed [67].

Intraoperative bleeding remains one of the most frequent complications during renal transplantation procedures, with 5.6% of patients requiring blood transfusion [68]. In the study by Reyna-Sepúlveda et al., which examined complications associated with renal allograft transplantation, the average estimated blood loss during surgery was 450 mL [69]. Intraoperative hypotension typically begins to occur when blood loss exceeds 30% of total blood volume.) Blood volume loss of 15–30% may lead to mild tachycardia and a slight decrease in blood pressure. Early signs of hypovolemia may be masked in anesthetized patients due to anesthesia-induced vasodilation and a blunted sympathetic response. Therefore, >30% blood loss intraoperatively is generally the threshold for clinically significant hypotension.

The absence of cutaneous manifestations (e.g., rash) and respiratory symptoms, and the non-temporal relationship of the beginning of hypotension to rATG administration, along with improvement following volume resuscitation, would help differentiate hemorrhagic hypotension from anaphylaxis.

Intraoperative myocardial infarction is another important differential to consider. Blood samples should be sent for cardiac biomarkers, and a TEE should be performed to assess for any wall motion abnormalities. If confirmed, appropriate management should be initiated promptly [70].

Intraoperative pulmonary embolism is rare but should still be considered and ruled out when suspected, using transesophageal echocardiography (TEE) [71]. The absence of right ventricular dysfunction makes this diagnosis less likely [72,73].

Similarly, hemolytic transfusion reactions and high spinal blocks are extremely uncommon during transplant procedures. Transplantation is typically performed in specialized centers with stringent quality controls, minimizing the risk of mismatched transfusions. Further, spinal anesthesia is rarely used for transplantation [74,75]. Although uncommon, these possibilities should be considered by clinicians in the intraoperative setting.

### 4.2. Other Clinical Syndromes to Consider in Differential Diagnosis of rATG-Associated Perioperative Anaphylaxis

There are three other conditions that can present with clinical findings similar to those of anaphylaxis: cytokine release syndrome (CRS), anaphylactoid reactions, and latex allergy or hypersensitivity to other drugs. A comprehensive comparison of these differential diagnoses is presented in Table 5.

#### 4.2.1. Cytokine Release Syndrome

CRS is a life-threatening condition that can occur after the administration of rATG. It involves widespread immune system activation, triggering a cytokine storm that intensifies the immune response and leads to various clinical presentations, including hypotension and respiratory failure. In addition to rATG, CRS has also been associated with the use of chimeric antigen receptor (CAR) T cell therapy, rituximab, dacetuzumab, nivolumab, and lenalidomide [76,77].

Symptoms may appear within minutes to several hours after exposure to a triggering agent. Early onset of symptoms is often associated with a greater risk of developing severe CRS [77,78,79]. Several cases of CRS following the administration of ATG in renal transplantation have been reported in the literature [12,80,81]. The onset of CRS has been observed both intraoperatively and postoperatively. The absence of elevation of serum tryptase and histamine and an increase in the inflammatory marker IL-6 in CRS facilitates the diagnosis.

#### 4.2.2. Non-IgE Mediated Anaphylactoid Reactions

Anaphylactoid reactions are non-IgE-mediated responses that involve the direct release of mediators from mast cells and basophils. The clinical manifestations of these reactions are similar to those of anaphylaxis [82]. These reactions can occur without prior exposure to the trigger and therefore do not require sensitization. They have been commonly reported following the administration of radiocontrast media and antibiotics [83]. The treatment of such reactions is also similar to anaphylaxis [84]. An anaphylactoid reaction has also been reported in association with rATG administration [85].

#### 4.2.3. Latex Allergy and Hypersensitivity to Other Drugs

Latex and drug allergies (neuromuscular blocking agents, barbiturates, and antibiotics) are among the most common causes of anaphylaxis during surgery. Their clinical manifestations are similar to those previously described for anaphylaxis [86]. Establishing a temporal relationship between exposure and symptom onset can help identify the specific trigger. Treatment includes immediate discontinuation or removal of the offending agent, followed by standard management of anaphylaxis.

### 4.3. Treatment Protocol

Treatment of anaphylaxis should begin immediately. The first step involves discontinuing the suspected allergen or triggering agent and administering IV epinephrine. During this period, anesthetic agent delivery, especially volatile anesthetic agents, should be reduced to acceptable levels. The fraction of inspired oxygen (FiO_2_) should be increased (>0.80) to help maintain oxygen saturation [87,88]. If the symptoms appear shortly after the start of rATG infusion, this temporal relationship can strongly help identify rATG as the likely cause, and the infusion should be stopped immediately. An appropriate dose of IV epinephrine should be administered [87,89].

According to several clinical guidelines, the recommended dose of intravenous (IV) epinephrine in such cases is 0.1 to 0.2 mg (100–200 mcg, IV bolus). In the event of cardiac arrest, 1 mg IV epinephrine should be administered every 1 to 2 min in accordance with ACLS protocol. If the systolic blood pressure falls below 50 mmHg, chest compressions are recommended, even in the absence of cardiac arrest [87,90]. In clinical settings, the standard vial concentration is 1 mg/mL. The dosage measurement and administration should be done very carefully, as it tends to have a very narrow safety window, and improper dosing can result in cardiac arrhythmias. If adequate symptom control is not achieved, a continuous infusion of 0.05–0.1 mcg/kg/min should be considered and titrated to response [87].

In typical clinical practice, antihistamines are administered along with vasopressors. This approach typically involves an H1 receptor antagonist such as diphenhydramine (25–50 mg, IV) or chlorpheniramine (10 mg, IV), in combination with an H2 receptor antagonist such as ranitidine (50 mg, IV administered slowly over 5 min). However, the supporting evidence for this practice is not well established [87].

In cases where bronchoconstriction is not responsive to epinephrine, a short-acting beta-agonist such as albuterol 2.5 mg is administered via nebulizer connected to an endotracheal tube and repeated as needed. Placing the patient in the Trendelenburg position or elevating the legs may help improve preload and can be used alongside the required fluid bolus as part of management [87]. A fluid bolus of 20 mL/kg IV bolus administered initially, and repeated as needed, is a common clinical practice. Patients with chronic kidney disease (CKD) may require additional consideration regarding volume management, but acute fluid resuscitation should take priority, while monitoring and adjusting fluid therapy as clinically appropriate. If hypotension does not respond to epinephrine, the use of additional vasopressors may be necessary. This can include a norepinephrine infusion dosed at 0.05–0.1 mcg/kg/min or vasopressin for cases of refractory vasoplegia, usually administered as an initial bolus of 1–2 IU (0.03 IU/kg), followed by a continuous infusion of 2 IU per hour [87].

In certain clinical situations, the use of intramuscular (IM) epinephrine may be considered. These include cases where the administration of rATG has been started well before the patient is transferred to the operating room, and the patient subsequently develops anaphylaxis while in the preoperative area or on the admission floor. IM epinephrine is also appropriate in situations where IV access is not present. The recommended dose of epinephrine is 0.3 to 0.5 mg (300–500 mcg), administered intramuscularly into the mid-thigh. If needed, the dose may be repeated up to three times at intervals of 5 to 15 min [89,91,92].

### 4.4. Proposed Management Algorithm

**Figure 3:**
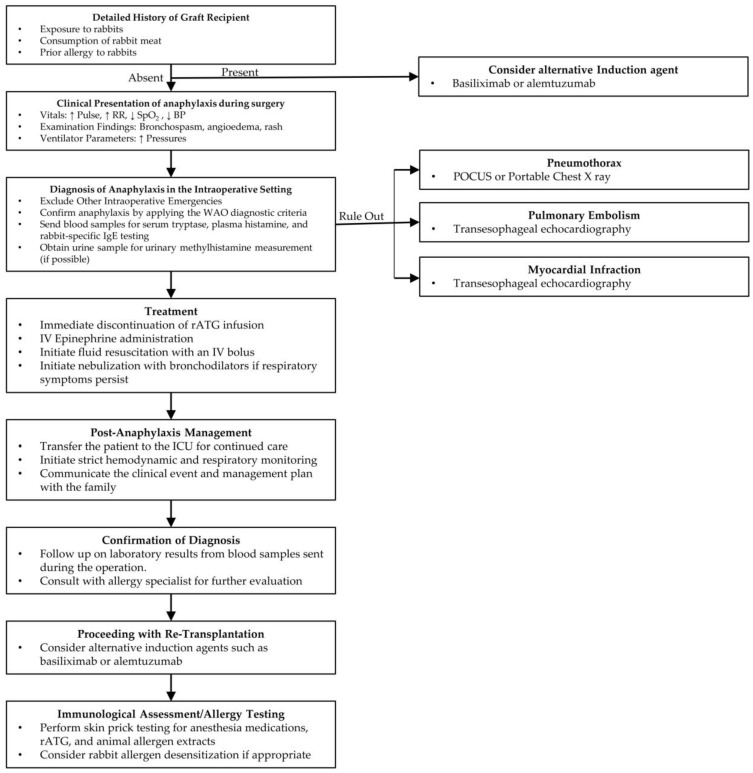
Proposed Management Algorithm for Anaphylaxis Associated with Rabbit Antithymocyte Globulin. Abbreviations: BP: Blood Pressure; ICU: Intensive Care Unit; IV: Intravenous; POCUS: Point of Care Ultrasound; rATG: Rabbit Anti-Thymocyte Globulin; WAO: World Allergy Organization.

Based on an analysis of previous case reports and our own experience with a similar case at our center, we have developed an algorithm that includes insights from transplant nephrology, surgery, anesthesia, and critical care. The management of anaphylaxis outlined in this algorithm is based on the clinical guidelines of the American Academy of Allergy, Asthma & Immunology/American College of Allergy, Asthma & Immunology Anaphylaxis Practice Parameters, and the Resuscitation Council United Kingdom (UK) guidelines for anesthetists [93,94]. Our proposed algorithm introduces several novel elements beyond existing guidance. It is designed specifically for the perioperative setting in renal transplantation, where rabbit antithymocyte globulin poses unique risks (Figure 3).

### 4.5. Prevention of Secondary Phase of Anaphylaxis

One of the risk factors for the development of biphasic anaphylaxis is the presence of severe initial symptoms [95]. These presentations of rATG-related anaphylaxis typically fall on the more severe end of the spectrum. Similarly, delay in administering and requiring multiple doses of an adrenergic agent (epinephrine) can increase the likelihood of a secondary phase [39,95]. The operating room setting can delay the timely detection of anaphylaxis; hence, it is important to diagnose anaphylaxis promptly and rapidly administer epinephrine. Although widely used, there is no compelling evidence to support the use of corticosteroids in the treatment of anaphylaxis or prevention of the secondary phase of anaphylaxis [96].

## 5. Post-Stabilization Decision-Making

Following stabilization of the patient after treatment for anaphylaxis, a decision must be made regarding whether to proceed with surgery. As there are no established guidelines for this situation, the decision is typically made on a case-by-case basis, depending on the severity of the reaction and the response of the patient to the treatment. The urgency of the surgical procedure may also influence the decision [87]. The challenge lies in balancing the immediate, life-threatening risk to a hemodynamically fragile patient against the potential loss of a viable, often scarce donor organ.

In a retrospective case–control study conducted by Sadleir et al., which analyzed 223 patients who developed intraoperative anaphylaxis, it was found that surgery was deferred in more than half of the cases [97]. Deferral was most common among patients who had more severe anaphylactic reactions [97]. This suggests that the severity of the allergic response significantly impacts the clinical course and decision-making during surgery. Deferring the transplantation surgery can lead to prolongation of the cold ischemia time of the allograft and can affect clinical outcomes. If the patient remains critically ill after an anaphylaxis episode, consideration should be given to place the kidney in a different recipient, or if possible, return the kidney to the organ procurement organization. In the case of a living donor, if the patient remains critically ill precluding living donor transplantation, auto-transplantation should also be considered. Saeed et al. reported a case of intraoperative anaphylaxis during a living donor kidney transplantation. The recipient was stabilized in the ICU followed by successful surgery using a different induction agent [10].

Most reported cases of anaphylaxis associated with ATG have presented with severe symptoms. The cases described by Kandil et al. and Navas-Blanco et al. required cardiopulmonary resuscitation (CPR) during the acute management [59,60]. In the majority of the reported cases, transplant surgery was deferred. However, in one case by Raval et al., the procedure was continued after the patient was stabilized and ATG was administered again in diluted form [61]. We recommend that the decision to proceed with surgery should be guided by the clinical condition of the patient. Given that the onset is often abrupt and severe, along with the high-risk context of renal transplantation, postponing surgery after stabilization may be the safest course of action.

We recommend that such patients be managed in the intensive care unit with continuous advanced monitoring and hemodynamic support. We recommend placement of an arterial line and central venous access if not already in place. Measurement of serial arterial blood gases (ABG) is also recommended until their condition improves and they are hemodynamically stable for surgery. During this period, the medical team should also meet with the family to explain the intraoperative events and to discuss the next steps. This observation period also allows for monitoring of a potential biphasic reaction. Studies have shown that one hour of observation has a negative predictive value (NPV) of 95.0%, while observation for six hours or more increases the NPV to 97.3% for detecting a biphasic reaction [98].

## 6. Diagnostic Workup for Anaphylaxis

The diagnostic evaluation of anaphylaxis includes measurement of serum histamine levels, tryptase levels, rabbit-specific IgE, urinary methylhistamine levels, and performing skin prick testing. These investigations help in supporting the diagnosis, identifying the trigger, and guiding future management.

### 6.1. Serum Histamine Levels

Serum histamine is a time-sensitive biomarker that is released immediately following exposure to an anaphylactic trigger. Its concentration peaks within the first 10 min and subsequently declines to baseline levels within the following hour [99]. Monitoring the temporal fluctuations in histamine levels may help establish a correlation with the triggering event, providing information regarding the potential causative agent.

In the study by Horiuch et al., which evaluated histamine for diagnosing perioperative hypersensitivity, it was observed that the sensitivity of serum histamine for diagnosing anaphylaxis decreased over time [100]. The primary drawback of this test is the very limited window for sample collection, as its effectiveness rapidly diminishes. However, despite these limitations, timely sample collection and conducting the test could still strengthen the diagnosis.

### 6.2. Serum Tryptase Levels

Serum tryptase is a serine protease released upon activation of mast cells, which can occur through either IgE-mediated or non-IgE-mediated mechanisms [101]. Following mast cell degranulation, blood levels of tryptase begin to rise, reaching a peak within 1 to 2 h. After peaking, tryptase levels decline following first-order kinetics, with a half-life of approximately 2 h [99]. This kinetic profile provides an adequate window for sample collection. Elevated serum tryptase levels can aid in the diagnosis of anaphylaxis or mast cell activation disorders. Serial measurement of tryptase levels can further increase the diagnostic accuracy in the assessment of anaphylaxis [102].

Serum tryptase has been reported to have a specificity greater than 97% for anaphylaxis, making it a highly reliable test for confirming the diagnosis [103]. This becomes particularly important in the context of intraoperative anaphylaxis, such as that associated with rATG administration, where clinical recognition may be complicated by the effects of anesthesia, surgery-related hemodynamic changes, and the presence of multiple potential causes of anaphylactic symptoms. The effectiveness of serum tryptase in distinguishing anaphylactic from non-anaphylactic events in the perioperative setting has been demonstrated in a study by Vitte et al. [104]. Elevated serum tryptase levels in several cases of rATG-induced anaphylaxis have been reported in the literature [10,38,60,61].

We suggest considering this test, as it can offer further support for the diagnosis. Serum tryptase has a longer time to peak compared to histamine levels, which makes it valuable even if blood sampling is delayed [99]. In the operating room, where physicians are often managing multiple priorities, this extended window allows for more flexibility in diagnostic assessment.

### 6.3. Urinary Methylhistamine Levels

Urinary methylhistamine (N-methylhistamine) is a primary metabolite of histamine, formed via the action of N-methyltransferase and excreted in the urine. Its presence serves as a useful surrogate marker for mast cell activation [105]. In a study conducted by Stephan et al., urinary methylhistamine levels were observed to increase by nearly 250%, with elevated concentrations still present after six hours [106].

However, the interpretation of urinary methylhistamine can be complicated in patients with impaired renal function. Altered kidney profiles may lead to delayed clearance or inconsistent excretion of solutes, potentially affecting metabolite levels [107]. In cases of oliguria or anuria, limited urine output may also result in insufficient sample volume, reducing the diagnostic utility of the test.

Despite these limitations, urinary methylhistamine remains a potentially valuable tool in supporting the diagnosis of mast cell-related disorders. Clinicians should interpret results within the context of deranged renal function and overall clinical presentation.

### 6.4. Skin Prick Testing

Skin prick testing (SPT) can be performed to identify the causative allergen. The procedure involves placing a drop of allergen extract solution on the skin, followed by pricking the skin to introduce the allergen, and then measuring the subsequent reaction [108]. In cases where transplant surgery has been deferred, SPT may help to confirm the source of the allergic reaction to ATG. We recommend the approach described by Brabant et al., who tested for latex, medications used during anesthesia induction, ATG, and commercially available standardized extracts for animal epithelia (rabbit, hamster, guinea pig, and horse) [38]. In their case, positive reactions were observed to rabbit and horse epithelia, helping to support the identification of the allergen. This approach can be used in guiding clinical decisions; however, it has important limitations. It cannot be performed immediately after surgery due to temporarily reduced skin reactivity (ideally, it should be done after 4–6 weeks); the urgency of reattempting kidney transplantation may not allow sufficient time for testing; and the availability and cost of specific allergen extracts may also present barriers [109]. Also, SPT may yield misleading results due to cross-reactivity among animal allergens [110].

### 6.5. Rabbit-Specific IgE Testing

Measurement of IgE antibodies specific to rabbit serum protein and dander may be a useful diagnostic tool in confirming the causative allergen. In the case reported by Saeed et al., intraoperative testing demonstrated a positive result for rabbit protein-specific IgE, supporting the diagnosis [10]. Similarly, Brabant et al. observed positive IgE findings when testing was performed several months later [38]. This shows that testing for IgE is informative both immediately after exposure and during follow-up assessments.

## 7. Proceeding with Re-Transplantation

Once the patient has been stabilized, weaned off the ventilator, and the diagnosis of anaphylaxis secondary to ATG has been confirmed, the next step is to proceed with the transplantation while avoiding the allergen, which in this case is rATG. An alternative induction agent should be used. Two induction agents that can be considered are basiliximab and alemtuzumab. These are monoclonal antibodies that function by blocking IL-2 receptors and targeting the cell surface protein CD52, respectively [111]. The successful use of basiliximab has been documented in cases of re-transplantation, and alemtuzumab also remains a viable alternative in such clinical scenarios [10,60]. The transplant team should proceed with heightened caution throughout the procedure.

Desensitization to ATG can be considered in cases where transplantation is delayed. However, given the availability of alternative agents such as basiliximab and alemtuzumab, this approach may not be routinely required. Also, there have been reports of anaphylaxis following desensitization [112,113]. For these reasons, we generally do not recommend this approach unless there is a strong clinical reason.

## 8. Limitations

This review and the proposed algorithm should be interpreted in light of several limitations. The available evidence on rATG-associated perioperative anaphylaxis is largely derived from isolated case reports, with a lack of prospective or randomized data. As a result, the true incidence and outcomes may be underestimated. The reliance on published cases also introduces potential publication bias, as severe or unusual reactions are more likely to be reported than milder or uneventful cases. The variability in clinical settings, anesthetic practices, and reporting standards further limits the generalizability of individual case findings. Finally, while our algorithm is informed by existing guidelines and multidisciplinary expertise, it should be considered a pragmatic framework rather than a definitive protocol.

## 9. Future Directions

Future directions involve improving our understanding of the pathogenesis of anaphylaxis related to rATG, identifying specific allergenic proteins, and improving pre-transplant screening strategies. Currently, testing for rabbit allergies is not routinely performed during pre-transplant evaluation, which may be due in part to the cost and limited availability of such testing in many centers. However, inquiring about known rabbit allergies, rabbit exposure, including petting, consuming, and occupational exposure (veterinary and laboratory settings) should be included in history taking.

Introducing skin testing protocols could represent a step forward. In addition, sequencing and identification of immune-related homologs may have the potential for identifying genetic associations with rATG-related anaphylaxis. The development of reliable biomarkers may also play an important role in diagnosing, preventing, and assessing the risk of anaphylaxis. The stratification of severity using such markers and novel therapeutic approaches could also be helpful in the future [114].

## 10. Conclusions

Anaphylaxis is a rare and potentially life-threatening complication associated with the use of antithymocyte globulin in renal transplantation. One of the contributing factors is the use of animal-derived components in antithymocyte globulin production, which may increase the risk of hypersensitivity reactions. Prompt recognition and multidisciplinary management of intraoperative anaphylaxis are important, as delays can result in poor outcomes. Clinicians involved in renal transplantation must remain vigilant about the risk of anaphylaxis, and a coordinated, team-based approach is important to achieve optimal patient outcomes.

## Figures and Tables

**Table 1:** Side effects of rabbit antithymocyte globulin.

Side Effects
Fever Diffuse hives/urticaria Leukopenia Thrombocytopenia Lymphopenia Hypotension Bradycardia Pulmonary edema Chest pain Cytokine Release Syndrome Anaphylactic shock Serum sickness Angioedema Disseminated Intravascular Coagulation Altered mental status

**Table 2:** Comparison of NIAID and WAO criteria for diagnosing anaphylaxis. NIAID: National Institute of Allergy and Infectious Diseases; WAO: World Allergy Organization; BP: Blood Pressure.

NIAID Criteria		WAO Criteria	
Anaphylaxis is likely when one of the following criteria is met:			
1.	Acute onset of symptoms with involvement of skin, mucosal tissue, or both, along with one of the following: Respiratory compromise or Low blood pressure or related symptoms	1.	Acute onset with skin or mucosal involvement and at least one of the following: Respiratory compromise Low blood pressure or end-organ dysfunction Severe gastrointestinal symptoms
2.	Two or more of the following after likely allergen exposure: Skin/mucosal symptoms Respiratory compromise Low blood pressure or related symptoms Gastrointestinal symptoms		
3.	Low blood pressure after known allergen exposure: Children: age-specific low BP or >30% drop Adults: <90 mmHg or >30% drop from baseline	2.	Rapid onset of hypotension, bronchospasm, or laryngeal involvement following exposure to a known or suspected allergen (within minutes to hours), even in the absence of typical skin symptoms.

**Table 3:** Summary of key reported cases of anaphylaxis secondary to antithymocyte globulin in the literature.

Study	Age/Sex	Ethnicity	Type of Donor	Indication for Transplantation	Past Medical History	Pre-Medication	Thymoglobulin Dose	Reaction Onset	Presentation	Management	Outcome
**Kandil et al.** **(2009)** [59]	39/F	NDA	Deceased donor	ESRD	Hypertension, colostomy, and reversal of colostomy	Methylprednisolone	125 mg IV	1–2 min after initiation	Absent pulse, hypotension, bradycardia, absent breath sounds on the left, wheezing on the right, decrease in end tidal CO_2_, and O_2_ saturation	CPR, atropine, epinephrine, mechanical ventilation, chest tube, pericardiocentesis	Discharged on day five; no information on retransplantation
**Brabant et al.** **(2017)** [38]	24/M	Caucasian	NDA	ESRD (spina bifida, neurogenic bladder with Bricker ileal conduit complicated by chronic renal failure)	Asthma, allergic rhinitis. Allergy/atopy: rabbits, pollens, house dust mites, latex, and vancomycin	NDA	12.5 mg/hour	Within 3 min	Hypotension, bradycardia, erythroderma, and bronchospasm	Thymoglobulin infusion stopped, resuscitation with external cardiac massage and adrenaline IV. Adrenaline infusion and terbutaline nebulization	Patient not transplanted as of report date
**Rafat et al.** **(2017)** [62]	43/M	NDA	Living donor	ESRD (Failure of previous transplantation)	Prior renal transplant	NDA	75 mg (at 4 AM)	Intraoperative (Total elapsed time since infusion not available)	Refractory hypotension (anaphylaxis as per WAO criteria for anaphylaxis)	Vasopressors, mechanical ventilation	Deteriorated postoperatively and expired after 24 h
**Navas-Blanco et al.** **(2018)** [60]	51/M	NDA	Deceased donor	ESRD	Uncontrolled hypertension, no documented allergies	Methylprednisolone, diphenhydramine	125 mg IV	Three minutes after initiation	Swelling around the head and neck, hypotension, low end-tidal CO_2_, tachycardia progressing to PEA arrest, and underfilled LV on TOE	CPR, IV epinephrine, norepinephrine infusion, mechanical ventilation, procedure aborted	Discharged on day five, developed chronic left-sided chest pain; no information on retransplantation
**Saeed et al.** **(2020)** [10]	67/F	Caucasian	Living donor	ESRD	Prior rabbit exposure (undocumented), no documented allergies	Diphenhydramine, acetaminophen, methylprednisolone, famotidine	75 mg IV	Within minutes	Hypotension, tachycardia, wheezing, and elevation of peak airway pressure	Stopped infusion, vasopressors started, IV steroids, plasmapheresis, and mechanical ventilation	Successful transplant 72h later with basiliximab induction
**Pyar et al.** **(2023)** [63]	58/M	NDA	Living Donor	ESRD (Possibly diabetic kidney disease)	Smoker, diabetes mellitus, multiple prior blood transfusions, and coronary artery disease	Premedication is not specifically mentioned; however, ATG administration followed transplant protocol	750 mg	After 10 h	Hypotension (3 h post-operatively), CVA secondary to anaphylaxis: altered mental state (GCS: 8/15), right hemiparesis, dysphagia, motor aphasia, right facial palsy, intravascular hemolysis, thrombocytopenia, acute liver injury, delayed allograft function, acute respiratory distress syndrome	Noradrenaline (3 h post-operatively), IM adrenaline, stopped ATG, started Basiliximab, steroids, hemodialysis, oxygen/NIV, PRP, insulin, fluid management, ACLS	Deteriorated on the 5th post-operative day. Anaphylactic reaction to the second dose of basiliximab. Patient expired after cardiac arrest during ETT insertion
**Raval et al.** **(2024)** [61]	22/M	NDA	Deceased donor	ESRD (Autosomal Dominant Polycystic Kidney Disease)	Hypertension; no documented allergy to latex or rabbits, and no known exposure to rabbits	Pheniramine maleate, acetaminophen, and methylprednisolone	75 mg	Within 2 min	Bilateral wheezing, which later progressed to a silent chest, Hypoxia, elevation of peak airway pressure, bradycardia, and hypotension	Thymoglobulin infusion stopped, IV adrenaline, fluid bolus, diphenhydramine, dexamethasone, and hydrocortisone. Salbutamol and budesonide via ETT. Ventilatory support adjusted. Trendelenburg position	Following stabilization, the procedure was continued. Renal transplant completed using double-diluted rATG
**Campbell et al.** **(2024)** [64]	12/F	Caucasian	NDA	ESRD (renal dysplasia)	No prior exposure to ATG or rabbits	Acetaminophen, methylprednisolone, diphenhydramine	NDA	Within minutes	Rash, hypotension, tachycardia, dyspnea	Epinephrine, corticosteroids, diphenhydramine	NDA

NDA: No data available; ESRD: End-stage renal disease; CPR: Cardiopulmonary resuscitation; WAO: World Allergy Organization; CVA: Cerebrovascular accident; GCS: Glasgow Coma Scale; ATG: Antithymocyte globulin; NIV: Noninvasive ventilation; PRP: Platelet-rich plasma; ACLS: Advanced Cardiovascular Life Support; rATG: Rabbit antithymocyte globulin; ETT: Endotracheal tube.

**Table 4:** Systematic summary of outcomes reported in published cases of rATG-associated anaphylaxis. Data were extracted from individual case reports (*n* = 8), summarized in Table 3.

Outcome	Number of Cases (*n*)	Percentage (%)
**Cardiovascular collapse (CPR required)**	4	50.0
**Procedure aborted/deferred**	3	37.5
**Procedure completed after stabilization**	4	50.0
**Reported mortality**	2	25.0
**Alternative induction agent used**	2	25.0

**Table 5:** Comparison of anaphylaxis, anaphylactoid reactions, and cytokine release syndrome.

Feature	Anaphylaxis	Anaphylactoid Reactions	Cytokine Release Syndrome
**Mechanism**	IgE-mediated (Type I hypersensitivity)	Non-IgE-mediated mast cell/basophil activation	Non-IgE-mediated mast cell/basophil activation
**Onset (after exposure)**	Minutes to hours	Minutes to hours	Hours to days (in some cases, it can occur within minutes)
**Symptoms**	Rash, hypotension, angioedema, bronchospasm	Similar to anaphylaxis	Fever, hypotension, hypoxia, organ dysfunction
**Laboratory Findings**	Elevated tryptase, elevated histamine, and elevated urinary methylhistamine measurement	Elevated tryptase, elevated histamine, negative IgE, and skin prick test	Elevated CRP, ESR, IL-6
**Diagnostic criteria**	NIAID/WAO Criteria	-	-
**Supportive Testing**	Serum IgE testing/Skin prick test	-	-
**Treatment**	Epinephrine, antihistamines, corticosteroids	Similar to anaphylaxis	Supportive care, IL-6 blocker (tocilizumab)
**Prognosis**	Resolves with treatment	Resolves with treatment	Can be severe and life-threatening

## Data Availability

All data used in this article are available in the references cited.

## Abbreviations

ATG	Antithymocyte globulins
CKD	Chronic kidney disease
CPR	Cardiopulmonary resuscitation
CRS	Cytokine release syndrome
EKG	Electrocardiography
FDA	Food and Drug Administration
ICU	Intensive care unit
IL-6	Interleukin-6
IM	Intramuscular
IV	Intravenous
NIAID	National Institute of Allergy and Infectious Diseases
NK	Natural killer
NPV	Negative predictive value
POCUS	Point-of-care ultrasound
rATG	Rabbit antithymocyte globulin
SPT	Skin prick testing
TOE	Transesophageal echocardiography
WAO	World Allergy Organization
